# Consuming *Gymnema sylvestre* Reduces the Desire for High-Sugar Sweet Foods

**DOI:** 10.3390/nu12041046

**Published:** 2020-04-10

**Authors:** Sophie Turner, Charles Diako, Rozanne Kruger, Marie Wong, Warrick Wood, Kay Rutherfurd-Markwick, Ajmol Ali

**Affiliations:** 1 School of Sport, Nutrition and Exercise, Massey University, Auckland 0745, New Zealand; S.Turner1@massey.ac.nz (S.T.); R.Kruger@massey.ac.nz (R.K.); W.Wood@massey.ac.nz (W.W.); 2 School of Food and Advanced Technology, Massey University, Auckland 0745, New Zealand; C.Diako@massey.ac.nz (C.D.); M.Wong@massey.ac.nz (M.W.); 3 School of Health Sciences, Massey University, Auckland 0745, New Zealand; K.J.Rutherfurd@massey.ac.nz; 4 Centre for Metabolic Health Research, Massey University, Auckland 0745, New Zealand

**Keywords:** gymnemic acids, pleasantness, liking, sweet taste, type 1 taste receptor

## Abstract

*Background*. Gymnemic acids, from the plant *Gymnema sylvestre* (GS), selectively suppress taste responses to sweet compounds without affecting the perception of other taste elements. The aim of this study was to investigate the effect of consuming a GS-containing mint on the desire to consume high-sugar sweet foods directly thereafter. *Methods*. This study utilized a single-blind, crossover design comparing the consumption of a mint (dissolving tablet) containing 4 mg of gymnemic acids with an isocaloric placebo in 56 healthy young men and women. Participants were given samples of their favourite chocolate (varied between 14–18 g; energy varied between 292–370 kJ) and were directed to rate on their hunger on 100-mm visual analogue scales 30 s prior to consuming high-sugar sweet food (chocolate). They then consumed the GS mint or placebo mint and rated their perceived pleasantness and desire for more chocolate on separate visual analogue scales immediately following consumption of the high-sugar sweet food before being offered up to five additional servings (and asked to rate hunger, pleasantness and desire to eat more chocolate between each ingestion period). *Results*. The number of chocolate bars eaten decreased by 0.48 bars (21.3%) within a 15-min period of consumption of the GS mint (*p* = 0.006). Desire to eat more of the high-sugar sweet food (*p* = 0.011) and pleasantness of the high-sugar sweet food (*p* < 0.001) was reduced after GS mint intake. Those who reported having a ‘sweet tooth’ had a greater reduction in the pleasantness of chocolate (*p* = 0.037) and desire to eat more (*p* = 0.004) after consuming the GS mint for the first serving of a high-sugar sweet food following the mint. *Conclusion*. Consuming gymnema-containing mints compared to placebo significantly reduced the quantity of chocolate eaten mainly due to a decrease in the desire and pleasantness of consuming it.

## 1. Introduction

Sugar intake is increasing globally due to changing diets, such as increased availability of sweet, processed foods [1]. A review of nationally representative surveys from 16 countries found adults consumed 13.5%–24.6% of their total energy from added sugar, which is far above the WHO recommended sugar intake level of 5%–10% of energy intake (approximately 12 teaspoons) per day [2,3]. A systematic review conducted by Te Morenga et al. [4] reported that increased body weight is associated with increased consumption of free sugars, with particularly strong links to sugar-sweetened beverage consumption.

Increasing added sugar in diets is one factor associated with a corresponding increase in body weight in adults [4]. By 2025, 18% of adult men and 21% of adult women worldwide are expected to be obese (have a body mass index (BMI) greater than 30.0 kg·m^−2^) [5]. In addition, the economic impact is significant as medical costs in the US were 36%–100% higher for obese populations than those with BMI in the healthy range [6]. Excessive sugar consumption is one of the public health issues that needs to be addressed through a range of different measures.

In Ayurvedic medicine (an Indian traditional health care system), the leaves of a large vine, *gymnema sylvestre*, are used to treat diabetes and dental caries, reducing obesity and inflammation [7]. *Gymnema sylvestre* has been reported to exhibit anti-diabetic properties as several constituents (gymnemic acids, gurmarin and gymnemasaponin V) act together to normalize blood sugar levels by decreasing plasma glucose and increasing insulin secretion by the pancreas and regeneration of pancreatic islet cells [7,8]. It has been found that chewing the leaves or brewing it as tea results in a temporary, reversible sweetness inhibitor [9,10]. This taste modulation has given *gymnema sylvestre* the reputation of being a “sugar destroyer” or “gurmar” in Hindi [7,11]. More recently, the active components from *gymnema sylvestre* have been identified and purified. Analysis of these components found sweetness suppression activity in humans was affected by a group of triterpene saponins called gymnemic acids [12,13,14]. In humans, gymnemic acids selectively suppress taste responses to sweet compounds without affecting the perception of other taste elements (salty, sour, bitter and umami) [14]. Sweet tastes are sensed by T1Rs (type 1 taste receptors), a group of G-coupled protein receptors found in taste bud cells and parts of the peripheral gastrointestinal tract [15]. Gymnemic acids are structurally similar to glucose molecules, and due to its anti-sweetness properties, acts as an antagonist and inhibitor of the T1R unit to produce the temporary suppression of the perception of sweet taste [10,16]. Further research is clearly needed to understand the effects of *gymnema sylvestre* and to consider its effectiveness as a potential tool against high global added sugar consumption and its effects. 

Gymnema has traditionally been prepared as a tea beverage either to be drunk or used as a mouth rinse [10,17]. Questions had previously been raised about whether the glucose-suppressing effects would persist after the leaves underwent food processing [9]; however, advancements in food processing have resulted in the development of several food products containing gymnema including sauces and breath mints [18,19]. Recent studies using formulated gymnema-containing products such as a dissolving tablet or “lozenge” found that consumption of gymnema in this form reduces both the intake and pleasantness of confectionary [20,21]. In a double-blind crossover study, consumption of a gymnema-containing “lozenge” reduced the desire for further servings of confectionary, in the two minutes prior to eating the next serving of confectionary [20]. This supported the hypothesis that the desire to consume more high sugar foods was not entirely dependent on the reduction in pleasantness experienced due to taste changes following consumption of the gymnema mint, but rather the reduction in desire was a result of reduced neural feedback to the reward response region of the brain [20,21]. 

From current evidence it is unclear whether any particular population subgroups would benefit from the anti-sweetness effects of gymnema more than others. For example, if the effect of gymnema may be more pronounced in higher refined sugar consumers or those who have been identified as supertasters. Supertasters (those who have a strong reaction to the bitter compound *6-*n*-propylthiouracil* (PROP)) have been shown to experience sweet foods more intensely than those who tasted PROP as less bitter (non-tasters or medium tasters) [22]. Supertasters therefore tend to prefer foods which are less sweet [23], whereas non-tasters or medium tasters reported a lower intensity of sweetness intensity for both sucrose and added sugars, and had a greater preference for sweet-tasting foods and an increased intake of added sugar [24]. As the intensity of sweet liking has been linked to increased consumption of these foods [25], PROP non-tasters or medium tasters may benefit more from using a product that inhibits the perception of sweet taste to reduce their intake. 

High BMI is associated with increased consumption of free sugars, as well as decreased perception of sweet taste and an increase in reported liking of high-sugar sweet foods [4,26,27]. To date however, no research has been conducted on the relationship between BMI and frequency of sugar food intake following the consumption of *gymnema sylvestre.*

Several sensory studies have suggested that men prefer more intense sweet-tasting food than women [28,29] and have a higher pleasantness rating when consuming sweet foods [26]. Conversely, other studies have shown that women are more likely than men to choose high sugar snack foods such as candy or chocolate to provide psychological and physiological comfort [30]. There are, however, no known studies to date that have explored gender differences in high-sugar sweet food consumption following sweet taste modulation.

The aim of the present study was to examine the effect of a sugar reduction tool (*gymnema sylvestre*) on the desire for high-sugar sweet food intake in different subgroups (gender, self-identified sweet taste preference, supertaster status and/or varying body composition). Identification of subgroups that may benefit from a product that creates an aversion to sugar-sweetened products by reducing desire and understanding the unique ways in which these groups respond to pleasantness, is important to gain an understanding of who may benefit from this tool.

## 2. Materials and Methods 

### 2.1. Study Participants

Fifty-six healthy adults (35.7% men and 64.3% women) participated in this study (mean ± SD age = 23.2 ± 5.7 years, range 18–47, BMI = 23.2 ± 3.1; Table 1). Participants were recruited using flyers distributed in and around the university campus and using email distribution lists to previous study participants. Inclusion criteria were age (18 and 50 years), and willingness to eat chocolate. Exclusion criteria included having coeliac disease or gluten intolerance (as the standard snack contained gluten), having diabetes, or having a pacemaker). Potential participants registered their interest in the study via an online pre-screening questionnaire (Qualtrics, 2019), to establish if they met the inclusion criteria, and captured their preferred chocolate selection from 15 popular choices (Table 2). Participants provided informed written consent prior to commencement of the study. Ethical approval was granted by the Massey University Human Ethics Committee: Northern (Application NOR 19/52). The trial was registered at Australian and New Zealand Clinical Trials (ACTRN12619001384145).

### 2.2. Research Design

This study utilized a within-subject, single-blind, crossover design, comparing the outcome of consuming a mint (dissolving tablet) containing 4 mg of gymnemic acids with an isocaloric placebo on the subsequent consumption of high-sugar sweet foods in healthy adult men and women. Chocolate was chosen as a proxy for high-sugar sweet foods to demonstrate this effect due to the standardisation of servings and high sugar content.

The study consisted of two visits (each approximately 1 h in duration) to the sensory laboratory, seven days apart. At the first visit to the sensory laboratory, the study procedure was explained, participants were presented with an information sheet and informed consent was gained. Participants then underwent body composition analysis, followed by the intervention test. Participants consumed one standard serve (22 g, 438 kJ) of wholegrain chips (Grainwaves) 45 min prior to sensory testing to reduce hunger. During this time, participants completed a questionnaire administered via online survey software (Qualtrics, 2019). Finally, sensory testing was conducted in sensory booths (sensory laboratory).

### 2.3. Measures 

#### 2.3.1. Anthropomorphic Measures

Weight was collected using calibrated scales. A stadiometer was used (single measure) to record participants’ height and BMI was calculated. 

#### 2.3.2. PROP Testing

Participants’ taster status was determined using 6-*n*-propylthiouracil (PROP), which is a compound that tastes strongly bitter to some individuals but is tasteless or weakly bitter to others. Participants were given a test paper impregnated with PROP (Precision Laboratories, United Kingdom) and asked to place it on their tongue for five seconds to allow it to become wet before removing it. Participants then rated the bitterness of the PROP on a labelled magnitude scale (LMS). Participants were classified as PROP non-tasters if they rated the taste intensity between “barely detectable” and “weak” on the LMS, a medium taster if they rated the intensity between “weak” and “strong” and a super taster if they rated the intensity between “strong” and “strongest imaginable”(i.e., the top of the LMS) [31].

#### 2.3.3. Questionnaire 

Demographic information including age, gender, pregnancy, breastfeeding status and ethnicity were collected. Health information including medications or supplements taken; diets followed for health, weight loss or cultural/religious reasons; participants’ satisfaction with their weight; and activity level were captured. Participants also self-reported whether they considered themselves to have a “sweet tooth”.

#### 2.3.4. Sensory Testing

**Figure 1:**
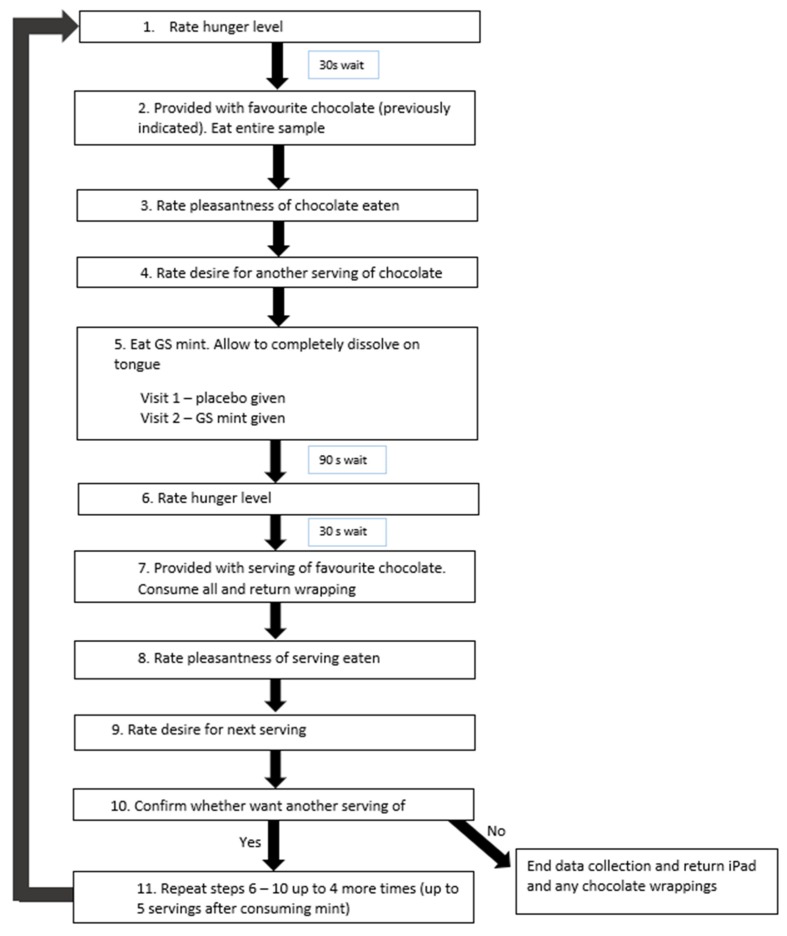
Flow diagram of research procedure.

Sensory evaluation was carried out in individual booths under red lights to mask the colour of the mints. Firstly, participants rated their hunger levels 30 s prior to consuming all of a standardized serving of their favourite chocolate (chocolates varied between 14–18 g; energy varied between 292–370 kJ; Table 2; Figure 1). Following consumption of the high-sugar sweet food (chocolate), participants rated the pleasantness perceived from eating the chocolate, and their desire for further servings. Thirdly, they received the mint and were asked to place it on their tongue, and suck on it until it completely dissolved. Participants waited 90 s after the complete dissolution of the mint and then again rated their hunger level, followed by consuming all of a second duplicate serving of chocolate. Again, the pleasantness of eating the chocolate, and desire to receive a further standard serving portion 90 s later, were recorded. 

Hunger, pleasantness of chocolate eaten and desire for another serving were each assessed using visual analogue scales (100-point VAS administered electronically). Participants rated their hunger between *“I am not hungry at all”* and *“I am extremely hungry”,* 30 s prior to the consumption of each chocolate serving. Pleasantness ratings were anchored by the statements *“Not at all pleasant”* and “*Very much pleasant”,* and the desire for eating another chocolate serving was rated from “*No, not at all”* to “*Yes, very much”* and were rated immediately following consumption of the food. Following the first chocolate eaten after the mint was consumed, participants were given the option to continue testing or to stop. If participants confirmed that they would like another serving of chocolate it was given, and the above process repeated for up to five servings after the mint was ingested. The time delay between each chocolate presented was two minutes after rating the previous chocolate. At any point the participant could opt out and say they did not desire any further chocolates.

Both the gymnema-containing mints (GS mint; “Sweetkick”) and the isocaloric placebo (same ingredients but without gymnema acids) were provided by the same manufacturer (Sweetkick, Nu Brands Inc; LA, California). At the first visit, all participants were given the placebo mint and they were all given the GS mint on their second visit. Results were collected electronically using the Compusense Cloud (Guelph, Canada) sensory software deployed on individual tablets (iPad, Apple Inc, Cupertino, California). 

#### 2.3.5. Statistical Methods

Data was analysed using R System for Statistical Computing (R Core Team, 2018). To describe the demographic variables of the participants, means and standard error of the mean were reported for continuous variables. Categorical data were reported as number of participants with the corresponding percentage. 

Assumption of normality were tested using the Shapiro-Wilk test and residual plots. Normally distributed data is presented as mean ± SD and geometric means (95% confidence intervals) and data not normally distributed is expressed as median (25^th^, 75^th^ percentiles). A *P* value less than 0.05 was considered statistically significant. Data from participants who refused subsequent servings of their favourite confectionery were treated as missing data, leading to an unbalanced repeated measures design. Linear mixed effects models fit by maximum likelihood was used to test for treatment group differences for mint type, serving and demographic variables (BMI, PROP taster status, gender and self-reported “sweet tooth” status) using contrasts. A random effect was included in the model to account for repeated measures. The multilevel linear model approach adopted for this analysis is advantageous because it explicitly models correlated scores from the same participant which is encountered in repeated measures and this addresses the assumption of independence of residuals. 

Chi-square analysis was used to determine significant differences in the number of consumers who rejected the next serving following the mandatory serving after tasting the mint. A paired *t*-test was carried out on data from the second serving. This was done to test the difference in mean ratings between the placebo and GS mint for participants’ desire for next serving and pleasantness of chocolate for the second serving immediately after participants had the mint. Effect sizes were calculated using the t-statistic and degrees of freedom from each test. An effect size of 0.1 indicated a small effect, a value of 0.3 indicated a medium effect and a value ≥0.5 indicated a large effect [32]. 

## 3. Results

### 3.1. Sweet Tooth and PROP Taster Status 

Table 3 shows the distribution of participants who self-identified as having a “sweet tooth” and their PROP taster group. Women identified as having higher sweet tooth than men (*p* = 0.018) and more women were in the “supertaster” group (*p* = 0.036). 

### 3.2. Effect of GS Mint on High-Sugar Sweet Food Consumption.

The total number of chocolate bars eaten at each lab visit decreased by 21.5% after consumption of the GS mint vs. placebo mint (from 2.25 ± 1.4 bars after the placebo to 1.77 ± 1.2 bars; *p* = 0.006). Furthermore, there was a reduction in energy intake by 21.4% following consumption of the GS mint (623 ± 414 kJ) compared to the placebo (792 ± 499 kJ; *p* = 0.007).

### 3.3. Desire for Next Serving, Pleasantness of High-Sugar Sweet Foods and Hunger Ratings

**Figure 2:**
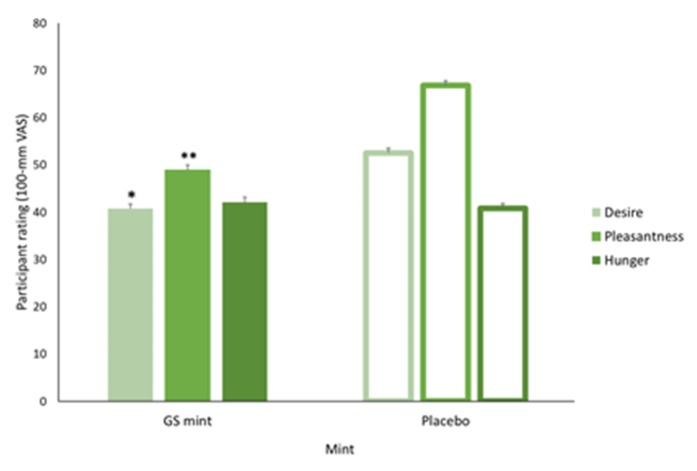
Participants’ ratings for desire (for more chocolate servings), pleasantness of eating and hunger for the first chocolate serving following consumption of gymnema and placebo mints (*n* = 56). Ratings were assessed using a visual analogue scale of 0–100 mm. The scale for desire for more ranged from 0 mm = *not at all* to 100 mm = *yes, very much*. Pleasantness ratings were anchored by 0 mm = *not at all pleasant* and 100 mm = *very much pleasant*. Hunger was rated from 0 mm = *I am not hungry at all* to 100 mm = *I am extremely hungry*. * significantly different to placebo (*p* < 0.05); ** significantly different to placebo (*p* < 0.001).

Participants’ ratings of their desire to eat the next serving, pleasantness of eating chocolate and hunger after tasting the GS and placebo mints respectively, are shown in Figure 2. Participants had 22.7% reduced desire for another serving of their chosen chocolate after ingesting the GS mint compared to the placebo (*p* = 0.011, *r* = 0.33). Similarly, pleasantness rating was 31.0% lower following ingestion of the GS mint (49 ± 2.9 mm) as compared to the placebo (67 ± 2.3 mm) and this was found to have a large effect on chocolate consumption (*p* < 0.0001, *r* = 0.61). In contrast, there were no differences in ratings of hunger between trials (*p* = 0.715).

### 3.4. Effect of Demographic Variables on Desire and Pleasantness

To determine whether BMI, gender, PROP taster status and self-reported sweet tooth status of participants had any effect on desire for next serving and pleasantness rating of the sweet food, a series of linear mixed models were fitted using each of the demographic variables and mint type as independent factors. Gender had an impact on pleasantness ratings as women had a lower average pleasantness rating (54.78 ± 2.42 mm) than men (63.61 ± 2.96 mm; *p* = 0.0266) following GS mint intake. However, gender did not impact the desire for next serving (*p* = 0.3727). Participants’ BMI did not cause significant differences in the desire for next serving (*p* = 0.5562) and the pleasantness ratings of the sweet foods (*p* = 0.9884). The PROP taster status of participants did not affect desire (*p* = 0.3228) or pleasantness (*p* = 0.5094) ratings. 

**Figure 3:**
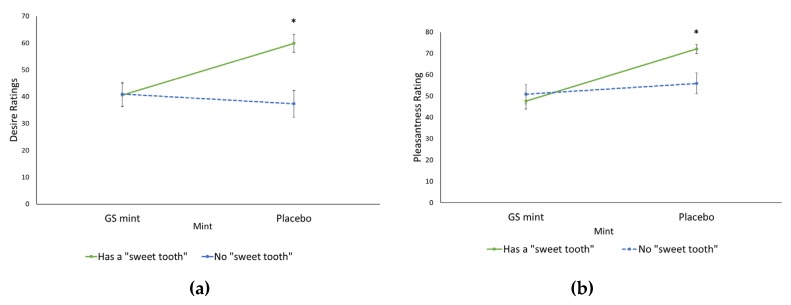
(**a**) Interaction between mint and sweet tooth status on participants’ rating of their desire for another chocolate serving. (**b**) Interaction between mint and sweet tooth on participants’ pleasantness rating of a high-sugar sweet food (chocolate). * significantly different to placebo (*p* < 0.05)

Sweet tooth status (“*Has a sweet tooth*” or “*No sweet tooth*”) had a significant effect on the desire ratings for both mints (Figure 3). Those with sweet tooth recorded a higher average rating for desire for subsequent (59.89 ± 3.37 mm) compared to those with no sweet tooth following consumption of the placebo mint (37.31 ± 4.99 mm; *p* = 0.0043). Participants’ desire ratings were similar for both groups following the GS mint, as the average rating for those with a sweet tooth was 40.62 ± 4.30 mm and those without sweet tooth 40.98 ± 4.45 mm, showing that those with a sweet tooth were more affected by the impact of the GS mint. Similarly, after consuming the placebo mint, participants with a sweet tooth recorded a higher average rating of the pleasantness of the sweet food chocolate (72.10 ± 2.18 mm) than those with no sweet tooth (55.95 ± 4.87 mm; *p* = 0.0377). In contrast, after ingesting the GS mint, the responses for pleasantness of the chocolate was similar for those with sweet tooth (47.69 ± 3.73 mm) and those with no sweet tooth (50.88 ± 4.51 mm; *p* > 0.05). 

**Figure 4:**
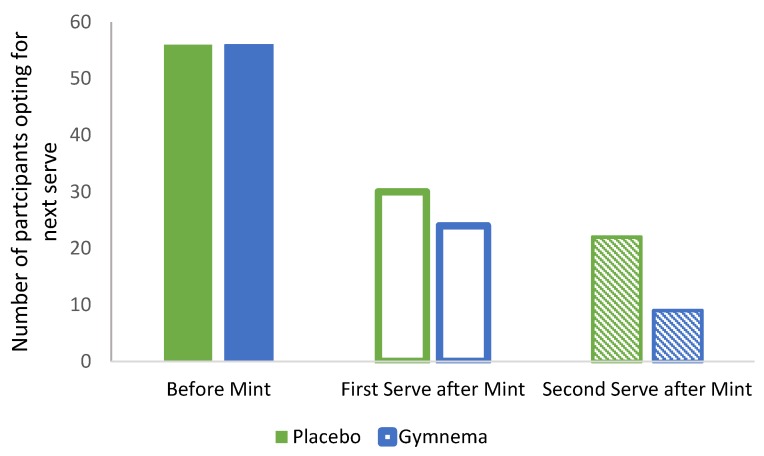
Number of participants opting for further servings of a high-sugar sweet food (chocolate) before and after mint consumption.

With each subsequent optional serving there was a steady decline in the number of participants who wanted another chocolate bar as shown in Figure 4. The second serving post consumption of either mint was significantly lower than the number of participants opting for another serve prior to consuming the mint (*p* = 0.038). This effect was more pronounced following intake of the GS mint with fewer participants opting for the next serve compared to the number who chose a subsequent serve after taking the placebo. The above pattern continued with the third and fourth serve after the mint, as following the placebo mint, 22 and 9 participants ate a third and fourth serve of chocolate respectively, whereas after the GS mint only 9 participants had a third serve and 6 participants had a fourth serve of chocolate. A paired t-test was used to investigate the relationship between the scale ratings (pleasantness, desire for more and hunger) with the rate of decline of the first optional serve (second serve post mint); pleasantness ratings decreased just after the GS mint was consumed (GS: 39.44 ± 3.68 mm, placebo: 62.90 ± 3.73 mm, *p* < 0.001). Consumption of the GS mint resulted in a significant decrease in the rating for desire for next serve (GS: 35.10 ± 4.21 mm; placebo: 47.46 ± 4.61 mm, *p* = 0.008).

## 4. Discussion

The primary finding of this study was that gymnema acid-containing mints reduced intake of sugar-sweetened foods. We found that participants ate 0.48 (21.3%) fewer servings of their favourite chocolate after consuming a GS-containing mint compared to placebo. Consumption of the GS mint also resulted in a 22.7% decrease in desire to eat high-sugar sweet foods and 31.0% reduced pleasantness of high-sugar sweet foods eaten but did not affect hunger. Furthermore, those who self-identified as having a “sweet tooth” had a greater reduction in both the pleasantness of eating high-sugar sweet foods and desire to eat more after consuming the GS mint compared to those who do not. There were no significant differences in amount of high-sugar sweet food eaten by BMI groups, gender or PROP taster status. 

### 4.1. Effect on Taste Receptors

The significant reduction in the amount of high-sugar sweet food eaten and the reported pleasantness experienced with eating indicates that the GS mint was effective in inhibiting the T1R2 + T1R3 heterodimer which senses sweet taste on the tongue [33]. These receptors are activated by sucrose but also other mono- and di-saccharides, sugar alcohols, and other small molecule sweeteners [16]. Since other taste dimensions are not affected; following suppression of the T1Rs, participants primarily tasted the other components of high-sugar sweet foods, i.e., the high cocoa content in dark chocolate which heightens the bitter taste. This disjunction between the expectation of the chocolate bar based on the label, brand and prior knowledge about how the product should taste does not align with the reality of the experience of the high-sugar sweet food they are eating, and of itself may reduce pleasantness and enjoyment of the product [34], and is supported by our data. 

### 4.2. GS Mint Reduces Acute Consumption of High-Sugar Sweet Foods (Acute vs Chronic Consumption)

Reduced consumption of high-sugar sweet foods following GS mint intake is consistent with previous findings [20,21]. Furthermore, a significant reduction in both pleasantness of high-sugar sweet food and the desire to consume more was found after consuming the GS mint in both the current investigation and Nobel et al. [20]. The resulting taste changes of high-sugar sweet foods experienced after consuming a gymnema-containing product are not the only contributing factor to this reduction in pleasantness and desire, as it has been reported that even prior to eating high-sugar sweet foods there is decreased activity in the reward response region of the brain following gymnema consumption [35]. fMRI imaging has shown that following consumption of a gymnema-containing product there was decreased neural recruitment in the areas of the brain associated with reward response (nucleus accumbens, precuneus, OFC, insula and caudate) [35]. In practice this may mean that consuming a GS mint may reduce ad libitum intake of other high sugar products even prior to the consumption of these products (decreased desire) and if they are eaten, they will not be as enjoyable (reduced pleasantness) to consume due to the sugar-suppression effects. In other words, as consumers will “know” that the GS mint will make the high-sugar sweet food not taste like it normally would, it will reduce their desire for the product (akin to learning effects), and so may subsequently reduce their intake of high-sugar sweet foods (in the longer term). 

### 4.3. Behavioural Aspects of Consuming GS Mints

Consumption of GS products result in a noticeable change in the way food tastes and may result in the perception that the other taste elements (e.g., bitter) are intensified [9]. This impacts on the enjoyment of high-sugar sweet foods eaten as participants reported a significant decrease (49 ± 2.9 mm vs. 67 ± 2.3 mm; Figure 2) in perceived pleasantness of eating high-sugar sweet foods following consumption of the GS mint; consistent with previous results wherein pleasantness rating was 22.2/100 mm lower after consuming the gymnema-containing product [20]. Therefore, consumption of sweet-tasting foods may be impacted following use of a GS-containing product. However, for this approach to be successful, consumers must have sufficient motivation to ingest the GS mint, knowing that it will prevent their enjoyment of high-sugar sweet foods. Consumers aiming to reduce their sugar intake that have reached the ‘preparation’ or ‘action’ stages of the transtheoretical (stages of change) model may find greater success with such an approach to restricting sugar consumption [36]. If, however, they have not reached these stages, they may not have sufficient motivation to voluntarily take flavour modulation products (e.g., GS mint) [36]. This is the case for other health behaviours such as smoking which also requires a high level of motivation to promote change, particularly when using interventions that result in temporarily reducing pleasantness and subjective wellbeing for long-term improvement [37]. Further research needs to be conducted to explore the relationship between motivation level and long-term success at reducing consumption of high-sugar sweet foods using GS mints. 

### 4.4. Effect of the GS Mint on Those with a “Sweet Tooth”

This study found that the effect of the GS mint was more pronounced in those who self-identified as having a “sweet tooth” than those who did not; i.e., GS mint intake led to greater reductions in pleasantness and desire ratings for high-sugar sweet foods in those with a sweet tooth. Reed [38] describes a “sweet tooth” as someone who prefers sweet foods, however preference for sweet foods may vary over time dependent on a number of modifiers including age, culture, mood, and accessibility. The present study observed that those who have a sweet tooth derived more pleasure from consuming sweet foods than those who do not (Figure 3). The brain has a strong pleasure response to sweet taste, so pursuit of pleasure and taste preferences are major drivers of sugar consumption [39,40]. Therefore, the GS mint may be more effective in reducing sugar intake in those with a self-proclaimed sweet tooth; however, more research, using a longer intervention period, will be required to investigate this assertion. 

### 4.5. Relationship between GS Mint and Gender

In this study we found gender had no effect on the amount of high-sugar sweet food consumed following the consumption of a GS mint. One possible explanation may be an interaction of the competing two factors that women are more likely to choose high sugar foods as a source of comfort [30], and therefore, on average, consume greater quantities of sugar, whereas men prefer a more intense sweetness than women [28,29]. Men reported higher non-significant mean ratings for pleasantness after the consumption of either the GS mint or placebo than women (*p* = 0.059). This is consistent with the findings that men report high-sugar sweet foods as more pleasant than women [26]. It is important to interpret this with caution as there were fewer men in this study (35.7% of participants), and men have been underrepresented in some [9,21,35] but not all [20] previous investigations on the sugar suppressive effects of gymnema. Overrepresentation of women in these studies may reflect consumer interest. 

### 4.6. Relationship between GS Mint Consumption and BMI

This experiment did not show a relationship between the consumption of the GS mint and BMI groups. Previous findings demonstrated that increased BMI was associated with decreased perception of sweet taste [41] and increased liking of high-sugar sweet foods [9]; therefore, suggesting that obese people would consume more high-sugar sweet food after consuming the GS mint. In this study no differences in chocolate consumption, desire or pleasantness were found between overweight/obese participants and normal weight participants. Others have suggested that fat consumption, rather than sugar, may play a larger role in obesity [26]. This would indicate consumption of gymnema-containing products may benefit those who have difficulty restraining their sugar intake, rather than a specific BMI group, but further studies are needed. 

### 4.7. Relationship between GS Mint Consumption and PROP Taster Status 

Current research suggests that PROP tasters (medium tasters and supertasters) perceive bitter and sweet-tasting compounds more intensely than other non-tasters and therefore are less likely to enjoy very sweet foods than non-tasters [28,42]. However, other studies have disputed this connection between PROP taster status and sweet food preference [43,44]. This study was consistent with the conclusion of the latter, finding no significant relationship between PROP taster status or the amount of a high-sugar sweet food eaten, pleasantness of the high-sugar sweet food nor desire for more. Despite the difference in preferred sweetness intensity, our findings suggest that this sugar reduction tool is unaffected by PROP taste status i.e., no effect of ‘supertaster’ status. However, anecdotally, some participants reported that the GS mint tasted bitter, which may relate to their PROP taster status, hence palatability of the mint may be higher among PROP non and medium tasters. There were more supertasters in this study compared to the 30% non-tasters, 50% tasters, and 20% supertasters distribution in the population [45]. But this distribution can vary based on gender [46,47]—females tend to be supertasters and frequently rated PROP bitterness more intensely compared to males—and ethnicity [48]. There were more females and Asians in the current study, and this may have contributed to the taster status distribution observed.

### 4.8. Study Limitations and Future Directions

One surprising finding was that several participants did not follow the expected trend of reduced consumption of high-sugar sweet foods after consumption of the GS mint, and in fact ate more servings despite the same participants giving the high-sugar sweet food low pleasantness ratings. We are speculating that this may be due to a “curiosity factor” as participants were unfamiliar with the sugar suppression effect of gymnema and wanting to experience the effect. We hypothesise that repeated exposure to this product would eliminate this “curiosity factor” as participants become accustomed to the taste modulation. 

This study was limited by the single-blinded approach. Participants received the placebo at the first visit and GS mint at the second. Participants were told they would receive two different products and instructed not to discuss the products with other participants due to slight differences in colour and taste of the placebo relative to the GS mint. Due to the distinct taste profile and difference in appearance, a conscious decision was made to provide the known prior to the unknown.

As with previous studies, men were underrepresented in this study. Further work in this area should target recruitment of men to explore any potential gender differences in consumption of sweet foods following intake of the GS mint.

To the best of our knowledge no evidence exists about the effects of long-term (chronic) intake of gymnema-products for its anti-sweetness effects. The expected length of sweet taste suppression for one GS mint is 30 to 60 min (based on manufacturer guidelines). Future research should explore the effects of chronic consumption of *gymnema sylvestre* products and the impact of consumption on free-living, non-diabetic adults, including whether the GS mint would be effective at altering sugar consumption habits if used whilst having a sugar craving, similar to the use of Nicotine Replacement Therapy to overcome a craving for a cigarette. Future investigations may also provide greater insights into the effect of the GS mint on those who have a “sweet tooth”. Initial results from this study gives evidence that this group may benefit more from the GS mint to control sugar intake as there was a larger decrease in pleasantness rating following consumption. Studies should screen for those who have a sweet tooth among a larger cohort. 

It is important to bear in mind the real-world applicability of these products. People generally consume a range of sweet foods, not just chocolate. Future research is needed to determine how much of an impact gymnema-containing products may have on sweet foods consumed outside of a laboratory environment, as consumers’ eating habits vary significantly, and they do not generally consume only one type of sweet food, so having access to a wider variety of sweet foods may alter their perceived pleasantness and desire for more sweet foods. Conducting qualitative research to understand participants’ experiences with gymnema-containing products would provide insight into how these laboratory findings may translate to everyday life and identify any issues surrounding taste, compliance and consumption of non-nutrient-rich sweet foods. 

## 5. Conclusions

This study aimed to investigate the impact of a *gymnema sylvestre-*containing mint on acute consumption of high-sugar sweet foods, as well as ratings of hunger, pleasantness and desire for more high-sugar sweet foods. The key finding was that the GS mint significantly reduced intake of high-sugar sweet foods compared to placebo and resulted in a decrease in the pleasantness and desirability rating of eating high-sugar sweet foods. Another key finding was that having a sweet tooth (relative to non-sweet tooth) resulted in a significant decrease in pleasantness and desire for more high-sugar sweet food after the GS mint compared to the placebo mint. The GS mint did not affect hunger ratings, nor was it impacted by BMI or PROP taster status. Future research is needed to assess the effects of longer-term consumption of *gymnema sylvestre* products on sugar consumption and adherence to such product use.

## Figures and Tables

**Table 1:** Participant characteristics.

Characteristic Presented as (mean ± SD) or *n* (%)	Total Group	Men	Women
**Gender**	56 (100)	20 (35.7)	36 (64.3)
**Age** (years)	23.2 ± 5.7	24.5 ± 7.6	22.4 ± 4.3
Median	21	23	21
Range	18–47	18–47	18–39
**BMI** (kg·m^−2^) mean ± SD	23.2 ± 3.1	24.4 ± 3.4	22.4 ± 2.7
**BMI group***n* (%)			
Underweight	3 (5.4)	0 (0)	3 (8.3)
Normal	39 (69.6)	12 (60.0)	27 (75.0)
Above normal (overweight + obese) ^1^	14 (25.0)	8 (40.0)	6 (16.7)
**Weight** (kg) mean ± SD	64.7 ± 12.1	74.9 ± 11.7	59.1 ± 8.0
**Ethnicity ^2^***n* (%)			
European	12 (20.7)	6 (26.1)	6 (17.1)
Asian	44 (75.9)	15 (65.2)	29 (82.8)
MELAA ^3^	2 (3.4)	2 (8.7)	0 (0)

^1^ The overweight (*n* = 12) and obese (*n* = 2) BMI groups were combined for analysis due to low numbers in each. ^2^ Participants were able to select multiple ethnic backgrounds and therefore the total is greater than 100%. ^3^ Middle Eastern, Latin American and African.

**Table 2:** Participant chocolate selection and nutrition information.

Chocolate	Participant Selection (*n*) ^1^	(%)	Weight (g)	Energy (kJ)	Sugar per Serve (g)	Sugar per 100 g (g)	Cocoa Content (%)
Nestle KitKat	18	32	17	370	8.6	50.5	22
Whittaker’s Almond Gold	12	21	15	360	5.2	34.6	33
Whittaker’s Creamy Milk	8	14	15	352.5	6.7	44.7	33
Whittaker’s Peanut Slab	6	11	15	333	7.2	48.0	33
Twix	3	5	14.5	308	7.0	48.0	25
Cadbury Moro Gold	2	4	17.5	327	7.3	48.6	26
Whittaker’s Dark Peppermint	2	4	15	342	7.8	52.1	50
Nestle Milky Bar	2	4	14.5	340	8.0	54.9	0
Cadbury Crunchie	1	2	15	292	10.3	68.7	26
Cadbury Flake	1	2	14	313	7.9	56.5	26
Snickers	1	2	18	370	9.3	50.6	25

^1^ Participant section of favourite chocolate in rank order.

**Table 3:** Sweet tooth and *6-*n*-propylthiouracil* (PROP) taster status.

Characteristic Presented as Mean ± SD or *n* (%)	Total Group	Men	Women
**Sweet tooth status *n* (%)**			
Has a “sweet tooth”	34 (60.7)	8 (23.5)	26 (76.5)
No “sweet tooth”	22 (39.3)	12 (54.5)	10 (45.5)
**PROP taster group ^1^**			
Non-taster/medium taster ^2^	17 (33.3)	9 (52.9)	8 (23.5)
Super taster	34 (66.7)	8 (47.1)	26 (76.5)

^1^ Data unavailable for 5 participants (8.9%); ^2^ Data combined for non-taster (*n* = 2) and medium taster (*n* = 15).
